# Leamos Juntos! Bilingual books support Latine parents’ Spanish language use during book-sharing interactions

**DOI:** 10.1017/S0305000925000182

**Published:** 2026-07

**Authors:** Alejandra Reinoso, Milton Guendica, Adriana Weisleder

**Affiliations:** Department of Communication Sciences and Disorders, Northwestern University, Evanston, Illinois, United States

**Keywords:** book-sharing, bilingual children, extra-textual talk, bilingual books, parent–child interactions

## Abstract

Book-sharing interactions expose children to diverse language input, yet most research on parent–child book-sharing has focused on monolingual parents reading monolingual books. This study investigated how Latine bilingual parents in the U.S. share different types of books with their children. Twenty-four Latine parents and their three- to five-year-old children shared a monolingual English-only book and a bilingual English-Spanish book. Parents used a higher proportion of total words and different words in Spanish when sharing the bilingual book than the monolingual book. They also engaged in more code-switching with the bilingual book than the English monolingual book. There were no differences in the number or diversity of words in English between book types. These findings show that bilingual books increase parents’ use of the home language (in this case Spanish) relative to books in the societal language, and suggest they may be one way of supporting children’s dual language development.

## Resumen

La lectura entre padres e hijos expone a los niños a nuevas palabras y formas de usar el lenguaje. La mayoría de las investigaciones sobre el uso de libros entre padres e hijos se han enfocado en familias monolingües que leen libros monolingües (generalmente en inglés). Este estudio investigó cómo los padres latinos bilingües en los EE.UU. leen diferentes tipos de libros con sus hijos. Veinticuatro madres o padres latinos y sus hijos de tres a cinco años compartieron un libro monolingüe en inglés y un libro bilingüe inglés-español. Los resultados muestran que las madres y los padres usaron una mayor proporción de palabras y de palabras diferentes en español al compartir el libro bilingüe que al compartir el libro monolingüe. También utilizaron más enunciados en que mezclaban el inglés y el español al compartir el libro bilingüe que al compartir el libro monolingüe. No hubo diferencias en la cantidad o la diversidad de palabras en inglés entre los dos tipos de libros. Estos resultados muestran que los libros bilingües aumentan el uso del lenguaje del hogar (en este caso, el español) en comparación a los libros monolingües, y sugieren que los libros bilingües pueden ser una forma de fomentar el desarrollo de lenguaje bilingüe en los niños.

## 1. Introduction

A majority of children around the world grow up learning two or more languages, and the number of bilingual children is growing even in countries with predominantly monolingual populations like the United States (U.S.). As such, it is a priority to identify practices that support bilingual children’s development and school success. Research shows that having strong oral language skills in both languages predicts bilingual children’s academic achievement, even when the home language differs from the language of schooling (Bedore et al., 2023; Giguere et al., 2024; López & Greenfield, 2004; Marchman et al., 2020). In addition, for immigrant children, learning and maintaining the home language is associated with stronger family relationships and better psychological adjustment, highlighting the social and emotional benefits of bilingualism (Suárez-Orozco et al., 2018; Weisleder et al., 2023). Indeed, most bilingual parents recognise the advantages of bilingualism and desire it for their children, yet they also note challenges in raising bilingual children, particularly when their home language is minoritised (Luo et al., 2023; Surrain, 2021).

One potential avenue for supporting the home language is through book-sharing (Fletcher & Reese, 2005; Noble et al., 2019; Read et al., 2020). Decades of research with monolingual families show that parent–child book-sharing is positively associated with young children’s language development and emergent literacy (Bus et al., 1995; Carmiol et al., 2024; Demir-Lira et al., 2019; Dickinson & Tabors, 2001). Two mechanisms that have been proposed for how book-sharing supports children’s language development are through the text of the book (Montag et al., 2015; 2020) and through parents’ extra-textual talk (Muhinyi et al., 2020; Read et al., 2023). However, most of these studies have focused on monolingual families sharing monolingual books. Thus, we know relatively little about book-sharing in bilingual families and, in particular, about how the text of the book might influence the language bilingual parents use during these interactions. Focusing on Latine^1^ families with preschool children in the U.S., the current study investigates bilingual parents’ extra-textual talk when reading a book with text only in English (English monolingual book) compared to a book with text in English and Spanish (English-Spanish bilingual book).

### 1.1. How book-sharing supports language and literacy development

Multiple mechanisms have been proposed for how book-sharing interactions support language and literacy development in children under the age of five. First, the text in children’s books includes language that is more diverse and complex than regular child-directed speech (Cameron-Faulkner & Noble, 2013; Montag, 2020; Montag et al., 2015). Therefore, when parents read the text in books, they expose children to more unique words and more complex sentence structures than in other contexts, which can facilitate growth in vocabulary and grammar (Demir-Lira et al., 2019; Huttenlocher et al., 2002; Jones & Rowland, 2017). The text in children’s books also mirrors academic language, which can be particularly important as children transition to formal education (Gámez, 2020).

Another way in which book-sharing interactions support language development is through extra-textual talk, which is talk that happens in the context of book-sharing but outside of reading itself (Mol et al., 2008; Read et al., 2023). Extra-textual talk is particularly supportive of children’s language learning in part because it can be tailored to the child’s experiences and interests, thus supporting their participation and learning from the book (Flack et al., 2018; Mol et al., 2008; Muhinyi et al., 2020). For instance, when adults use interactive reading strategies, such as talking about the story in ways that connect to the child’s life experiences, they can facilitate children’s word learning and story comprehension. It can also encourage children to participate verbally, thereby advancing their expressive language skills (Dickinson & Tabors, 2001; Hindman et al., 2008).

Although these two aspects of book-sharing have often been studied separately, there is also some evidence that the text of the book influences the characteristics of parents’ extra-textual talk. One study found that parents’ extra-textual talk during book-sharing exhibited higher lexical diversity than talk in other contexts (Hoff-Ginsberg, 1991). Another study showed that parent talk was more syntactically complex when parents shared books with their child than when they engaged in toy play, and that syntactic complexity was highest when the books included more complex grammatical constructions (Noble et al., 2018). Together, this suggests that the linguistic characteristics of the text in children’s books may be reflected in parents’ extra-textual talk. But what happens when bilingual parents share books with their children? Does the text of the book influence the characteristics of parents’ extra-textual talk regardless of the language parents are using? Does the language of the text influence what language parents use in their extra-textual talk? The current study aims to shed light on these topics.

### 1.2. Book-sharing in bilingual families

Because bilingual families have the option of reading in two languages, one important factor for understanding the characteristics of parent extra-textual talk in these families is the language they use during book-sharing. Recent studies suggest that reading practices in bilingual households vary according to the family’s dominant language (Avelar et al., 2024; Gonzalez-Barrero et al., 2021; Read et al., 2021). For example, a study of French-English bilingual families in Canada found that parents reported owning more books and reading more to their children in the dominant than in the non-dominant language, regardless of whether that language was English or French (Gonzalez-Barrero et al., 2021). However, this may be different for bilingual families in the U.S., given the asymmetrical status of English and other minoritised languages. In particular, since schooling in the U.S. takes place primarily in English, it is possible that parents choose to use English during book-sharing due to the belief that this will help prepare their children for school, even when English is not their dominant or preferred language.

Indeed, a recent study of Spanish-English bilingual families in the U.S. found that although parents’ language dominance influenced their shared reading practices, there was an overall bias for reading in English (Avelar et al., 2024). As expected, parents who were English dominant read more frequently in English than in Spanish, yet parents who were Spanish dominant read about equally in English and Spanish. Notably, all parents, regardless of dominance, owned more books in English than in Spanish, likely due to the greater availability of these books in the U.S. Thus, although language dominance is clearly one factor that influences bilingual parents’ language use during shared book-sharing, other factors – such as the sociolinguistic context and the language of the book – are also likely to play a role.

### 1.3. Code-switching in bilingual families

Another phenomenon that is important to understand about book-sharing interactions in bilingual families is the extent to which they engage in code-switching or language mixing. Studies in the U.S. and Canada suggest that code-switching is common among bilingual parents, but there is wide variability in the extent to which they do this, which may be related to the contexts of interaction (Bail et al., 2015; Byers-Heinlein, 2013; Kremin et al., 2022). Although many bilingual individuals code-switch as a natural response to their sociolinguistic environment (López et al., 2023), some parents may use code-switching – particularly during book-sharing – as a purposeful strategy to support their children’s learning and comprehension. For example, bilingual parents may seek to support their children’s bilingual vocabulary by labelling the same referent in both languages (e.g., *Look at the dog! ¿Te gusta el perro? ‘*Do you like the dog?’) or by embedding words in one language within sentences in the other language (e.g., *Mira el dog!* ‘Look at the dog!’). Scholars have proposed that teachers’ use of bilingual book-sharing practices such as these can be effective in expanding bilingual children’s vocabulary and semantic organisation in both languages (Lugo-Neris et al., 2010). Indeed, a recent study of bilingual preschool children’s word learning from books showed that older children learned more words in their non-dominant language when those words were embedded in passages in the dominant language (Read et al., 2020). Therefore, the use of code-switching during book-sharing may be an effective way for parents to support the home language, particularly as children become more dominant in the language of schooling, as it allows them to leverage children’s knowledge in one language to support the other.

A small number of studies have investigated the use of code-switching in the shared reading practices of bilingual families. In a qualitative study of Latine families in the U.S., parents mentioned intentionally alternating between English and Spanish when sharing books to create opportunities for biliteracy development, typically using their home language to assist with initial comprehension and following with an English translation (Perry et al., 2008). Another qualitative study of Korean-English bilingual families found that parents used code-switching during shared readings to make connections between common meanings in each language (Song, 2016). Some case studies have also found that bilingual parents code-switch when sharing picture books to emphasise and clarify vocabulary across languages (Kabuto, 2010; Moody et al., 2021). Finally, a recent study of French-English bilingual parents found that parents code-switched rarely when sharing a book in their dominant language but did so more often when sharing a book in their non-dominant language or a bilingual book, as well as when the parent and child had a different dominant language (Quirk et al., 2024). Taken together, these studies suggest that parents may use code-switching to scaffold children’s learning and comprehension during book-sharing interactions. The current study expands on these investigations and asks whether bilingual children’s books may provide unique opportunities for families to engage in code-switching and to use their home language.

### 1.4. Bilingual children’s books

Bilingual books have been growing in popularity, and there is growing interest in understanding whether they can be useful in supporting bilingual children’s language and literacy development (Benitez et al., 2022; Brouillard et al., 2022; Naqvi et al., 2013; Read et al., 2020; Semingson et al., 2015). Scholars have argued that by presenting the same narrative side by side in two languages, along with relevant illustrations, bilingual picture books can help support children’s comprehension of the story, learning of word-meaning mappings, and recognition of printed words across both languages, as well as increase metalinguistic awareness (Naqvi et al., 2013; Semingson et al., 2015; Zaidi, 2020). Indeed, studies suggest that bilingual preschoolers are able to learn novel words in two languages when exposed to them through bilingual books in a laboratory context (Brouillard et al., 2022; Read et al., 2020). Moreover, one study with kindergarteners in Canada found that bilingual children who were read bilingual books had greater gains in early literacy skills after an 11-week book-reading program than bilingual children who were read only monolingual books (Naqvi et al., 2013). This suggests bilingual books are a promising tool for supporting bilingual children’s language development. However, less is known about how bilingual books are used by parents when reading with their young children at home.

A small number of case studies have examined parent–child interactions in bilingual families sharing bilingual storybooks, and these show that parents incorporate both languages in their extra-textual talk (Kabuto, 2010; Moody et al., 2021). More recently, a study with French-English bilingual parents in Montreal examined parents’ extra-textual talk while reading books in their dominant language, books in their non-dominant language, and bilingual books (Quirk et al., 2024). This study found that parents used similar amounts of extra-textual talk across all three book types. However, parents produced more words in the non-dominant language when reading the bilingual book and the non-dominant language book than when reading the dominant-language book. They also produced more code-switches when reading the bilingual and non-dominant language book than when reading the dominant-language book. These findings suggest that reading bilingual books boosts parents’ talk in the non-dominant language relative to reading a dominant-language book, but not relative to a non-dominant language book.

Before generalising these results, however, it is important to consider the specific context in which this study took place. Quirk et al. (2024) conducted their study in Montreal, where both French and English are widely used and have relatively high status (Kircher, 2014). Public schooling, as well as early childhood education, is available in both French and English, and both French and English literacy materials are also widely available. This is a fairly unique context in North America and contrasts with the sociolinguistic context of the U.S., where, although there is no official national language, English functions as the de facto majority language, and all other languages (including Spanish, the second most widely spoken language) are marginalised in most institutional and educational settings (Lippi-Green, 2012). In addition, although the participants in the Quirk et al. study varied in their language dominance (some were dominant in French and others in English), their proficiency was generally high in both languages. Therefore, there is a continued need to understand bilingual parents’ book-sharing practices in different sociolinguistic contexts.

## 2. Current study

The current study seeks to understand how sharing an English-Spanish bilingual book versus an English monolingual book influences Latine parents’ extra-textual talk and particularly their use of Spanish, the home or heritage language. Children who are Hispanic and/or Latine make up 25% of the U.S. population aged 17 and under, and this number is expected to increase to almost 40% by 2050 (Chen & Guzman, 2021; Forum on Child and Family Statistics, 2023). Although most Latine children live in homes where Spanish is spoken, many Latine parents report challenges in supporting their children’s Spanish language development (Coba-Rodriguez & Jarrett, 2022; Surrain, 2021). Given the potential role of book-sharing for supporting young children’s language learning, the goal of the current study is to examine whether bilingual books with text in English and Spanish influence parents’ use of Spanish in their extra-textual talk to a greater extent than books with text in English only, as this would suggest English-Spanish bilingual books are a promising tool for supporting the development and maintenance of the home language. We chose to compare English-Spanish bilingual books to English-only books, and not to Spanish-only books, because English-only books are much more common in the U.S.; therefore, we were interested in understanding how bilingual books, which are growing in popularity but much less studied, would compare to what is essentially the societal default.

There are multiple ways in which bilingual books with text in English and Spanish could influence Latine parents’ language use during book-sharing interactions. First, the dual-language text is likely to make both languages more accessible and thus facilitate parents’ use of Spanish in their extra-textual talk relative to a book with text only in English. In addition, because the text in children’s books tends to be more lexically diverse than regular child-directed speech, the Spanish text in bilingual books may scaffold parents’ use of more different words in Spanish than they would use without the text’s support. Finally, bilingual books could influence Latine parents’ use of code-switching. Given that mixing languages is frowned upon in many U.S. contexts and even within some Latine communities (Mata, 2022), it is possible that seeing both Spanish and English in a published book will signal to parents that using both languages is acceptable and result in greater code-switching. Further, bilingual books may facilitate parents’ use of code-switching as a language learning strategy (Kabuto, 2010). In sum, bilingual books may create unique opportunities for Latine parents to use Spanish that may not be present when sharing English-only books.

Our specific research questions (RQs) were:

- RQ 1: Do Latine parents use more Spanish in extra-textual talk when sharing an English-Spanish bilingual book with their child compared to an English monolingual book? We predicted that parents would use more Spanish in extra-textual talk when sharing the English-Spanish bilingual book versus the English monolingual book.
- RQ 2: Do Latine parents use more lexically diverse extra-textual talk in Spanish when sharing an English-Spanish bilingual book versus an English monolingual book? We predicted that parents would use more different Spanish words in extra-textual talk when sharing the English-Spanish bilingual book versus the English monolingual book.
- RQ 3: Do Latine parents use more code-switching in extra-textual talk when sharing an English-Spanish bilingual book versus an English monolingual book? We predicted that parents would use more code-switched utterances in extra-textual talk when sharing the English-Spanish bilingual book versus the English monolingual book.

Finally, although we did not originally set out to examine the role of language dominance or proficiency, given recent studies showing that language dominance affects parents’ language use during shared book reading (Gonzalez-Barrero et al., 2021; Quirk et al., 2024), we conducted an exploratory analysis asking whether parents’ self-reported Spanish or English proficiency differentially affected their use of Spanish and/or code-switching across book conditions.

## 3. Method

### 3.1. Participants

Twenty-four parent–child dyads (22 mothers, 2 fathers) participated in the current study. Parents and their children were recruited from multiple sources, including a paediatric primary care clinic near a largely Latine populated area in a large Midwestern city in the U.S. as well as via a participant registry for child language studies and social media advertisements. Families were invited to participate in a study of parent–child book-sharing observations over Zoom. IRB approval was obtained from Northwestern University and Rush University Medical Center. All parents completed informed consent at enrolment.

All parents met the following eligibility criteria: identified as Hispanic or Latine, were residing in the U.S., and were English- and/or Spanish-speakers. Children were all typically developing based on parent reports. Child age ranged from three to five years (*M* = 3;9; *SD* = 6 months). As shown in Table 1, parents had a range of education levels, with most having a college degree. Parents were also asked to report how well they understand, speak, read, and write in English and Spanish, each on a 5-point Likert scale (1 = not well at all, 5 = extremely well). As parents’ ratings were comparable across items, we chose speaking as our indicator of proficiency. On average, parents’ proficiency was high for both English (M = 4.25, SD = 1.15) and Spanish (M = 4.04, SD = 1.27), yet there were some differences across parents. Thus, for further analysis, parents were classified into a *lower Spanish proficiency* group if they had scores of 1–3 (speaks not at all to moderately well) and into a *higher Spanish proficiency* group if they had scores of 4–5 (speaks very well to extremely well). The same criteria were used to classify parents into a *lower English proficiency* and a *higher English proficiency* group.

**Table 1.** Participant demographics (*n* = 24) Table 1. Long description.

	*n* (%)
Child Gender	
Male	10 (41.7)
Female	14 (58.3)
Parental Education	
Less than HS or some HS	4 (16.7)
HS equivalent or Some college	4 (16.7)
College degree	11 (45.9)
Graduate degree	4 (16.7)
Missing	1 (4.2)
Parent language proficiency^1^	
Lower Spanish proficiency	7 (29)
Higher Spanish proficiency	17 (71)
Lower English proficiency	3 (12.5)
Higher English proficiency	21 (87.5)

1 Lower Spanish/English proficiency = spoke Spanish/English “not well at all,” “slightly well” or “moderately well.” Higher Spanish/English proficiency = spoke Spanish/English “very or extremely well”.

### 3.2. Materials

#### 3.2.1. Questionnaires

Parent language proficiency was assessed using a modified version of the English language proficiency questionnaire used in the Early Childhood Longitudinal Study – Birth Cohort (ECLS – B) (Cabrera et al., 2006), as described above and shown in Table 1. Parents also completed a separate demographic questionnaire which asked questions about family and language background. Parents indicated in which language they preferred to speak and read to their child on a 5-point Likert scale (1 = All English, 2 = More English than Spanish, 3 = Equally in English and Spanish, 4 = More Spanish than English, and 5 = All Spanish). Responses were grouped into: All or Mostly English (1 and 2), All or Mostly Spanish (4 and 5), and Both English and Spanish (3); see Table 3.

#### 3.2.2. Books

The books used in this study were selected based on the following criteria: (1) was a commercially available children’s book, (2) was appropriate for children 3–5 years of age, (3) was a storybook with a main character, not a concept book, and (4) was available in two language formats: monolingual English-only and bilingual English-Spanish. In addition, we selected books with themes and characters not specific to Latine culture, as this was the topic of a separate study (with a separate, non-overlapping group of participants). The English monolingual books were written only in English; the English-Spanish bilingual books were translation books, such that they included the English text alongside Spanish translations. Native Spanish speakers reviewed the English-Spanish bilingual books to ensure the translations were accurate and the Spanish usage was natural and appropriate.

We selected two sets of monolingual and bilingual books. The first set of books were: *How Do I Love You?* (English version) and *How Do I Love You? ¿Como te Quiero?* (English-Spanish bilingual version) (Marion Dane Bauer, 2008, 2014), which are about a parent explaining their love to a child. The second set of books was: *My Friends* (English version) and *My Friends. Mis Amigos* (English-Spanish bilingual version) (Taro Gomi, 2005, 2006), about a girl who describes learning different skills (e.g., climbing, jumping) from her friends, many of them animals. These two books were similar in length and amount of text (see Table 2).

**Table 2.** Linguistic characteristics of the text for each book Table 2. Long description.

	English monolingual books		English-Spanish bilingual books	
	My Friends	How Do I Love You?	My Friends *Mis Amigos*	How Do I Love You? *¿Como te quiero?*
Number of sentences	17	17	34	34
Mean sentence length (words)	9.06	8.83	8.68	8.31
Total number of words	154	159	295	299
Number of unique root words	47	67	99	134

Each dyad received an English-only version of one book/story (*My Friends* or *How Do I Love You?*) and a bilingual English-Spanish version of the other book/story (*My Friends. Mis Amigos.* Or *How Do I Love You? ¿Como te Quiero?*). Story was counterbalanced across participants, such that half of the dyads (n = 12) were randomly assigned to receive the English-only version of *My Friends* and the bilingual version of *How Do I Love You?*, while others received the bilingual version of *My Friends* and the English-only version of *How Do I Love You?* Reading order was also counterbalanced across participants such that half of the dyads (n = 12) were randomly assigned to read the English-Spanish bilingual book first and others to read the English-only book first.

### 3.3. Procedure

The study sessions were all completed remotely via Zoom. Prior to the scheduled session, parents were mailed the two books and asked to complete online questionnaires. During the remote session, parent–child dyads met with a bilingual researcher who ensured that participants were in a comfortable and quiet space in their home and positioned in close proximity to their computer or phone camera and microphone. Dyads were first given a brief explanation of the book-sharing task. Then, parents were asked to read the two books that they received in the order that they were assigned (e.g., “First you will read *How Do I Love You?* And then you will read *My Friends. Mis Amigos.*”). No instructions were given as to which language should be used, just to “share the book as you typically would.” After addressing any questions, the researcher informed the parent that they would be on the call with their camera and audio off for the duration of the interaction. The researcher also asked the parent to let them know when they finished sharing the two books with their child. Once the book-sharing interaction was complete, the researcher turned their camera and audio back on and ended the session by asking the parent and child which book they preferred and thanking them for their time. Families were gifted the books that they read and received compensation upon completion of the session.

### 3.4. Transcription

Each Zoom interaction was video- and audio-recorded with parent consent. All video-recordings were transcribed by the first author (a Latine, Spanish-English bilingual PhD student) and a trained research assistant who also identified as Latine and Spanish-English bilingual. Transcription was completed using the Spanish-English bilingual guidelines for the Systematic Analysis of Language Transcripts (SALT) (Miller & Iglesias, n.d.). The first author and the research assistant completed extensive transcription training following the SALT online modules (https://www.saltsoftware.com/training) and guidance from an experienced PhD student, requiring approximately 15 hours of training and practice. Sessions were assigned to one of the two transcribers, and all transcripts were subsequently reviewed and verified by the first author (who was also one of the transcribers). During the verification process, transcripts were reviewed for errors in transcription and coding.

Each dyad had two transcripts: one for the English monolingual book and one for the English-Spanish bilingual book. Transcription began when dyads started to read or speak about the book and ended when dyads stopped reading or speaking about the book. Because sessions were conducted in the home, there was variability between families in the extent to which off-topic behaviours and conversations took place. Given that our focus was specifically on extra-textual talk, we excluded any speech that was considered non-book talk (e.g. off-topic conversations, directives for discipline) as well as utterances where parents read directly from the book. In a few cases, parents translated the text of the book into the other language. This was considered a form of reading, and these utterances were also excluded from our measures of extra-textual talk.

### 3.5. Language annotation and coding

To calculate the amount of extra-textual talk in each language, transcripts were annotated at the utterance and the word level. Specifically, a [spa] code was inserted for utterances with *only* Spanish words, a [eng] code for utterances with *only* English words, and a [mix] code for utterances with *both* English and Spanish words (that is code-switches within utterances). Subsequently, individual words in mixed utterances were annotated so we could analyse the language of each word. Words or utterances for which the language was ambiguous (e.g., ok, uh-oh) were coded as ambiguous if we could not make a clear phonetic distinction to determine the language (e.g., oʊˈkeɪ in English versus oˈkei in Spanish); ambiguous words were not included in the counts for either language. Cases in which the parent switched languages between utterances were coded as [interES] or [interSE] depending on if the switch was from English to Spanish, or Spanish to English, respectively. Utterances were not marked as code-switches if the switch happened from an English-only or Spanish-only utterance to a mixed utterance. For further detail on how code-switches were coded, see the coding guide and examples in OSF https://osf.io/msuyq/

### 3.6. Measures of parental extra-textual talk

The annotated transcripts were analysed using SALT (https://www.saltsoftware.com/), which calculated: (a) the number of word tokens in each language, (b) the number of word types in each language, and (c) the number of code-switches. We then calculated the proportion of word tokens and types that were in Spanish by dividing the number of Spanish tokens/types by the total number of tokens/types in both languages. For code-switches, we calculated the proportion of code-switches by dividing the number of code-switches by the total number of utterances.

#### 3.6.1. Word tokens

The number of word tokens (number of total words [NTW] value in SALT) was used as a measure of the amount of extra-textual talk. Word tokens were calculated across languages and also separately for English and Spanish. The proportion of word tokens that were in Spanish was then calculated by dividing the number of Spanish tokens by the sum of the Spanish and English tokens. We refer to this measure as the *proportion of Spanish tokens.*

#### 3.6.2. Word types

The number of different word types (number of different words [NDW] value in SALT) was used as our primary measure of lexical diversity. Word types were calculated in SALT by computing the number of unique word roots. Word types were computed across languages and separately for English and Spanish. The proportion of word types that were in Spanish was calculated by dividing the number of Spanish types by the sum of the Spanish and English types. We refer to this measure as the *proportion of Spanish types.*

Because the number of different word types in a speech sample is correlated with the length of the sample, we also calculated type-token ratio (TTR) as an additional measure of lexical diversity. The type-token ratio in Spanish (Spanish TTR) was calculated by dividing the number of Spanish word types by the number of Spanish word tokens. This is a more conservative measure of lexical diversity that controls for the total number of words in the sample (Malvern et al., 2004). However, TTR is also influenced by sample length and can over-correct such that shorter samples’ lexical diversity is inflated (Montag et al., 2018). Although the problems with TTR can be reduced by using a moving window approach (Covington & McFall, 2010), it was not possible to use that approach here given that there was a mix of English and Spanish in the interactions, and we were interested in calculating a separate TTR for each language. Therefore, we use TTR as a secondary measure of lexical diversity.

#### 3.6.3. Code-switches

Code-switches included all instances in which parents switched languages either between utterances (e.g., *Ese es un caballo. Is he big or small?* ‘That is a horse. Is he big or small?’) or within utterances (e.g., *Te quiero como la bee.* ‘I love you like the bee.’). The proportion of code-switches was computed by dividing the number of between- or within-utterance code-switches utterances by the total number of utterances.

### 3.7. Reliability

Sessions were assigned to one of the two transcribers, and all transcripts were subsequently reviewed and verified by the first author. During the verification process, transcripts were reviewed for errors in transcription and coding. To determine reliability in transcription, 20% of the video interactions (*n* = 5) were transcribed by both transcribers. The Intraclass Correlation Coefficient (ICC) was then calculated for each measure of interest (utterances, tokens, types). The ICC is used to assess the consistency of measurements made by two or more independent raters and is appropriate for assessing inter-rater agreement for numerical data (Shrout & Fleiss, 1979). It is a more powerful reliability statistic than Cohen’s Kappa when using a continuous measurement, such as the number of words. We performed a two-way mixed, single score ICC (ICC(3,1)), which is appropriate when the same coders code every observation, as was the case for our transcribers. For the English monolingual book, the ICC was .99, .99, and .99 for utterances, tokens, and types, respectively. For the bilingual book, the ICC was .99, .94, and .99, respectively. These indicate very high consistency in the measures derived from transcripts that were independently transcribed by the two transcribers. Any disagreements were discussed until reaching consensus, and consensus transcripts were used for analysis.

To assess the reliability of the language annotation and coding, a different 20% of transcripts (*n* = 5) were double coded by the two transcribers. The ICC was calculated for English types and tokens, Spanish types and tokens and code-switches. For the English monolingual book, the ICC was .99 and .95 for English and Spanish tokens, respectively, .99 and .94 for English and Spanish types, and 1.00 for code-switches. For the bilingual book, the ICC was .99 and .97 for English and Spanish tokens, .97 and .96 for English and Spanish types, and .98 for code-switches. As above, these values indicate a very high degree of consistency in the measures obtained from the two transcribers. Any disagreements were discussed and resolved through consensus.

### 3.8. Analytic approach

Our primary measures were the proportion of word tokens in Spanish (proportion of Spanish tokens), the proportion of word types in Spanish (proportion of Spanish types), and the proportion of code-switched utterances. We chose to use proportions for our primary measures to control for potential differences in overall talkativeness unrelated to our questions of interest – which are about differences in parents’ use of Spanish between books. In secondary analyses, we also compared the number of Spanish and English word tokens, the number of Spanish and English word types, the number of code-switched utterances, and the Spanish TTR.

The distributions of each measure were examined for normality using Shapiro–Wilk normality tests. None of the dependent measures were normally distributed. Therefore, Wilcoxon signed-rank nonparametric tests were used to compare each dependent measure between the bilingual and the English monolingual books. This test compares the ranks of the differences between pairs and is a non-parametric alternative to a paired t-test when the data is not normally distributed. Effect sizes were computed using *r* = abs(*Z*)/√*N* for the Wilcoxon signed-rank tests (Fritz et al., 2012). Because the data were not normally distributed, we report the median and interquartile range for descriptive statistics. All statistical analyses were completed using RStudio Version 4.2.2 (R Core Team, 2022).

## 4. Results

Before presenting results from the book-sharing interaction, we describe analyses of the questionnaire data, which revealed differences in parents’ preferences for speaking versus reading with their child (see Table 3). Specifically, whereas ten parents reported that they spoke primarily with their child in Spanish, only four reported reading primarily with their child in Spanish. Conversely, whereas seven parents reported speaking primarily with their child in English, eleven reported reading primarily with their child in English. This is consistent with previous studies of Spanish-English bilingual parents in the U.S. (Avelar et al., 2024) and suggests a bias towards reading in English even among parents whose predominant language is Spanish. These patterns help contextualise the main results of this study.

**Table 3.** Primary language in which parents speak and read to child Table 3. Long description.

Primary language in which parent:	*N* (%)
Speaks to child	
Mostly or only English	7 (29.1%)
Mostly or only Spanish	10 (41.6%)
Equally English and Spanish	7 (29.1%)
Reads to child	
Mostly or only English	11 (45.8%)
Mostly or only Spanish	4 (16.6%)
Equally English and Spanish	9 (37.5%)

### 4.1. Preliminary analyses

Data from the book-sharing interactions were first analysed to examine whether there was an effect of story (*How Do I Love You?* vs *My Friends*) and book order (monolingual first versus bilingual first). We compared the proportion of Spanish word tokens, Spanish word types, and code-switches by story and order using nonparametric Wilcoxon Rank Sum Tests (for two independent samples). There were no significant differences in the proportion of Spanish word tokens, word types, or code-switches between the two English monolingual books (*How Do I Love You?* and *My Friends.)* or the two English-Spanish bilingual books (*How Do I Love You?¿Como te Quiero?* and *My Friends. Mis Amigos.*), suggesting there were no effects of story on our primary variables of interest.

When examining potential order effects, we found that there was a significantly higher proportion of Spanish word tokens and word types for the English-Spanish bilingual book when the English-Spanish bilingual book was shared second (*Mdn* = .60 for tokens, *Mdn* = .55 for types) than when it was shared first (*Mdn* = .06 for tokens, *Mdn* = .14 for types), *W* = 32, *p* = .02, CI: 95% [−0.80, −0.02] for tokens, *W* = 33, *p* = .02, CI: 95% [−0.65, −0.04] for types. Although this order effect is interesting and may be worthy of future study, it was unexpected, and thus we do not discuss it further here. Importantly, since order was counterbalanced in our study such that half of the participants (*n* = 12) were randomly assigned to read the English-Spanish bilingual book first and half (*n* = 12) to read the English-Spanish bilingual book second, this should not affect our main results.

Finally, given the wide variation in children’s ages (3–5 years), we examined whether the child’s age was associated with any of our measures. Child age was not significantly correlated with the proportion of Spanish tokens (*r* = −0.075, *p* = 0.61), proportion of Spanish types (*r* = −0.081, *p* = 0.59), or the proportion of code-switches (*r* = −0.047, *p* = 0.75) used by parents. Given that child age was not associated with our main dependent variables, we do not adjust for age in our analyses. We have added graphs of these correlations in our Supplementary Materials: https://osf.io/msuyq/.

### 4.2. Primary analyses

On average, book-sharing interactions lasted approximately 12 minutes in total. For the English monolingual book, the average interaction length was 5 minutes and ranged from 1–9 minutes. For the English-Spanish bilingual book, the average length was 7 minutes and ranged from 2–18 minutes. Prior to analysing parents’ extra-textual talk, we examined how parents read the text of the books. When reading the English-Spanish bilingual book, most parents (66.6%) read the text in both languages, three parents (12.5%) read the text only in English, and two parents (8.3%) read the text only in Spanish. In contrast, when sharing the English-only monolingual book, most parents (83.3%) read the text only in English, while one parent (4.2%) read the text in English and then translated it into Spanish, and two parents (8.3%) did not read the text in English and instead only translated the text into Spanish. This shows the expected pattern – that parents are more likely to read the book in English and Spanish when the book includes text in both languages – but also reveals a diversity of bilingual reading strategies for both books.

Next we turn to our primary analyses, regarding parents’ extra-textual talk (ETT). Table 4 shows descriptive statistics for the number of English and Spanish word tokens, word types, and code-switches in parent ETT as a function of book type, as well as the proportion of Spanish word tokens and types and the proportion of code-switches. As shown in this table, parents used more English than Spanish overall during the book-sharing interaction. This is consistent with the results of parents’ self-reported preferences for speaking and reading to their child, shown in Table 3. We return to these findings in the discussion.

**Table 4.** Descriptive values of parent extra-textual talk measures by language and book type Table 4. Long description.

	English monolingual book			English-Spanish bilingual book		
Variable	Mdn	Inter-quartile Range	Range	Mdn	Inter-quartile Range	Range
Word tokens						
English	74.5	427	0–623	102.5	288	0–1279
Spanish	7	90.25	0–350	34.5	104	0–488
Proportion^1^	0.20	0.78	0–1	0.30	0.78	0–1
Word types						
English	49	127.75	0–187	59	111.75	0–234
Spanish	3	45	0–118	26.5	42.75	0–154
Proportion^2^	0.20	0.69	0–1	0.31	0.60	0–1
Code-switches						
Total	1	8.97	0–34	7.5	11.28	0–34
Proportion^3^	0	0.11	0–0.33	0.09	0.15	0–0.31

1 Spanish word tokens divided by total word tokens.

2 Spanish word types divided by total word types.

3 Number of code-switched utterances divided by total number of utterances.

### 4.3. Effect of book type on the amount of parental extra-textual talk in Spanish

**Figure 1.**
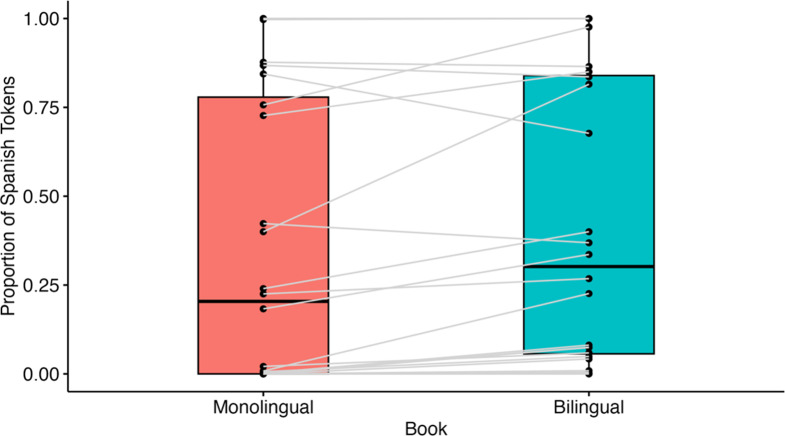
Proportion of Spanish word tokens by book type. Box plots showing the proportion of Spanish tokens for the English monolingual (left) and English-Spanish bilingual (right) books. Each box represents the inter-quartile range for that condition; the horizontal black line within each box represents the median, and the whiskers represent the minimum and maximum values. Dots represent individual participants, and the connecting lines show the change from the English monolingual to the English-Spanish bilingual book for each participant. Figure 1. Long description.

Parents used a higher proportion of Spanish word tokens in extra-textual talk when sharing the English-Spanish bilingual book (*Mdn* = .30) than when sharing the English monolingual book (*Mdn* = .20), Z = −2.55, *p* = 0.01, CI: 95% [−0.11, −0.01], with a medium effect size (*r* = .52*)*, see Figure 1. This difference was driven by parents using more Spanish word tokens when sharing the English-Spanish bilingual book (*Mdn* = 34.5) than when sharing the English monolingual book (*Mdn* = 7.0), Z = −2.55, *p* = 0.011, while there were no significant differences in their use of English word tokens when sharing the English-Spanish bilingual book (*Mdn* = 102.5) relative to the English monolingual book (*Mdn* = 74.5), Z = .365, *p* = 0.715.

### 4.4. Effect of book type on the lexical diversity of parental extra-textual talk in Spanish

**Figure 2.**
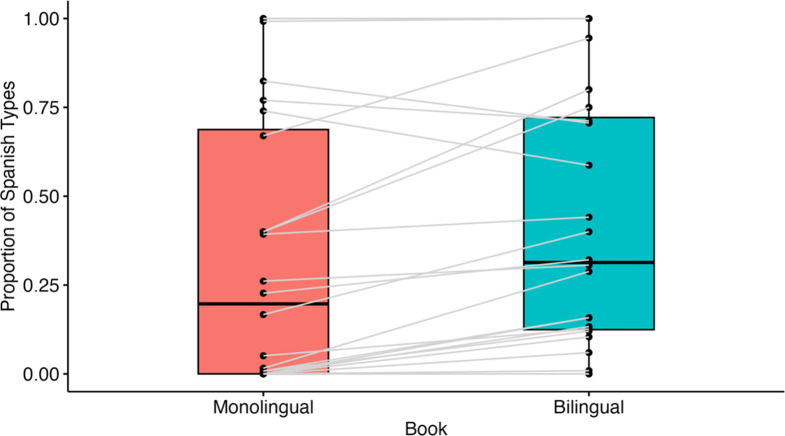
Proportion of Spanish word types by book type. Box plots showing the proportion of Spanish types for the monolingual (left) and bilingual (right) book. Each box represents the inter-quartile range for that condition; the horizontal line within the box represents the median, and the whiskers represent the minimum and maximum values. Dots represent individual participants, and the connecting lines show the change from the English monolingual to the English-Spanish bilingual book for each participant. Figure 2. Long description.

Parents also used a higher proportion of Spanish word types when sharing the English-Spanish bilingual book (*Mdn* = .36) than when sharing the English monolingual book (*Mdn* = .19), Z = −2.85, *p* = 0.004, CI: 95% [−0.21, −0.05], with a medium effect size (*r* = .58*)*, see Figure 2. Again, this difference was driven by parents using more Spanish word types when sharing the English-Spanish bilingual book (*Mdn* = 26.5) than when sharing the English monolingual book (*Mdn* = 3.0), Z = −3.15, *p* = 0.002, while there were no significant differences in their use of English word types when sharing the English-Spanish bilingual book (*Mdn* = 59.0) relative to the English monolingual book (*Mdn* = 49.0), Z = .400, *p* = 0.689. Given that the number of different words in a speech sample increases as a function of the number of tokens (e.g., Montag et al., 2018), and that parents produced more Spanish word tokens when sharing the English-Spanish bilingual book than the English monolingual book, it is possible that the difference in Spanish word types resulted from this increase in the number of tokens rather than being an independent effect of the English-Spanish bilingual book on lexical diversity. To address this possibility, we conducted an additional analysis using type-token ratio (TTR). Parents’ TTR in Spanish was higher when sharing the English-Spanish bilingual book (*Mdn* = .69) than when sharing the English monolingual book (*Mdn* = .45), but this difference did not reach statistical significance, Z = −1.74, *p* = 0.082, CI: 95% [−0.46, −0.03], and had a small effect size (*r* = .35).

### 4.5. Effect of book type on the number of code-switches in parent extra-textual talk

**Figure 3.**
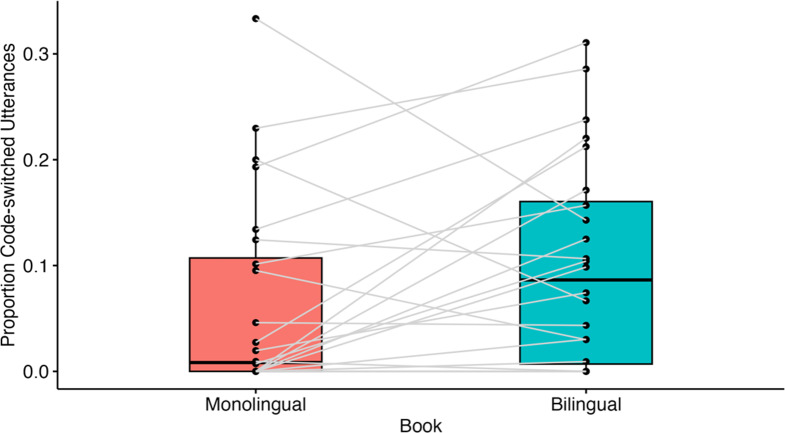
Proportion of code-switched utterances by book type. Box plots showing the proportion of code switches for the monolingual (left) and bilingual (right) book. Each box represents the inter-quartile range for that condition; the horizontal line within the box represents the median, and the whiskers represent the minimum and maximum values. Dots represent individual participants, and the connecting lines show the change from the English monolingual to the English-Spanish bilingual book for each participant. Figure 3. Long description.

Finally, although parents used a higher proportion of code-switches in extra-textual talk when sharing the English-Spanish bilingual book (*Mdn* = .08) than when sharing the English monolingual book (*Mdn* = .008), this difference did not reach significance, Z = −1.79 *p* = 0.07, CI: 95% [−0.10, −0.004], and had a small effect size (*r* = .36*)*, see Figure 3. On the other hand, there was a significant difference in the frequency of code-switches, with parents using a higher number of code-switches when reading the bilingual (*Mdn* = 7.5) than the monolingual (*Mdn* = 1) book, Z = −2.0 *p* = .04, CI: 95% [−1.25, −5.93], small effect size *r* = .41.

These results show differences in the degree to which parents use Spanish in their extra-textual talk. In addition, we examined differences in the number/proportion of parents who used each language when reading each of the books. We classified each parent based on whether they used any (i.e., >0) extra-textual words in English/Spanish when reading each book. Most parents (58%) used English and Spanish when reading both the monolingual and the bilingual book; however, the number of parents that used each language varied by book type ( $\chi^{2}$ = 20.41, *p* < .001). Specifically, 63% of parents used both Spanish and English in their extra-textual talk when reading the English monolingual book, while 79% did so when reading the English-Spanish bilingual book. These differences were due to more parents using Spanish when reading the English-Spanish bilingual book: 71% of parents used Spanish (and 92% used English) in their extra-textual talk when reading the English monolingual book, while 92% of parents used Spanish (and 88% used English) when reading the English-Spanish bilingual book. Thus, parents were more likely to use both languages, and specifically Spanish, when reading the English-Spanish bilingual than the English monolingual book.

### 4.6. Exploratory analyses: Moderating effect of parental Spanish proficiency

Our primary analyses suggest that reading an English-Spanish bilingual versus an English monolingual book affects parents’ use of Spanish in extra-textual talk. However, one possibility is that book type has a differential effect on parents’extra-textual talk as a function of their language dominance or proficiency (Quirk et al., 2024). Although our study was not initially intended to ask this question, we addressed this possibility in an exploratory analysis. We first calculated a difference score for each participant and each dependent measure between the English-Spanish bilingual book and the English monolingual book (e.g., proportion Spanish tokens in the English-Spanish bilingual book – proportion Spanish tokens in the English monolingual book). Then, we conducted Mann–Whitney U nonparametric tests (for two independent samples) with continuity correction to compare these difference scores between the lower and higher Spanish proficiency groups. Effect sizes were computed using Cliff’s Delta (2*U*/*n*_A_*n*_B_ – 1) for the Mann–Whitney *U* tests (Goedhart, 2016).

**Figure 4.**
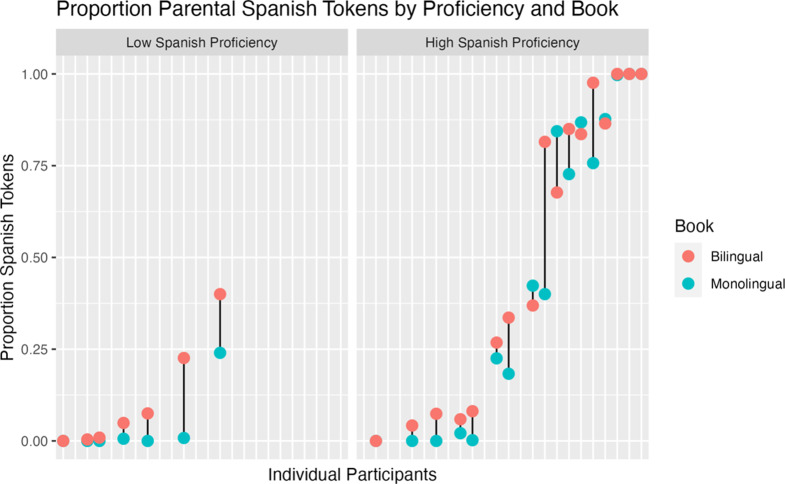
Proportion of Spanish word tokens by proficiency and book. Proportion of Spanish tokens used by each participant when sharing the English-Spanish bilingual book (red dots) versus the English monolingual book (blue dots), grouped by participants who had lower Spanish proficiency (*n* = 7) (left panel) and higher Spanish proficiency (*n* = 17) (right panel). The black lines connecting the red and blue dots illustrate the difference in the proportion of Spanish tokens between books for each participant. Figure 4. Long description.

Results did not reveal significant differences between the higher (*Mdn* = .043) and lower Spanish proficieny (*Mdn* = .038) groups in the difference score for proportion of Spanish word tokens *W* = 45, *p* = 0.37, CI: 95% [−0.11, 0.04], Cliff’s delta = −.2. Figure 4 shows that: (1) parents who were more Spanish proficient used a higher proportion of Spanish word tokens overall, and (2) across both proficiency groups, a majority of participants used a higher proportion of Spanish tokens when sharing the English-Spanish bilingual book than when sharing the English monolingual book. Thus, Spanish proficiency did not moderate the effect of book type.

**Figure 5.**
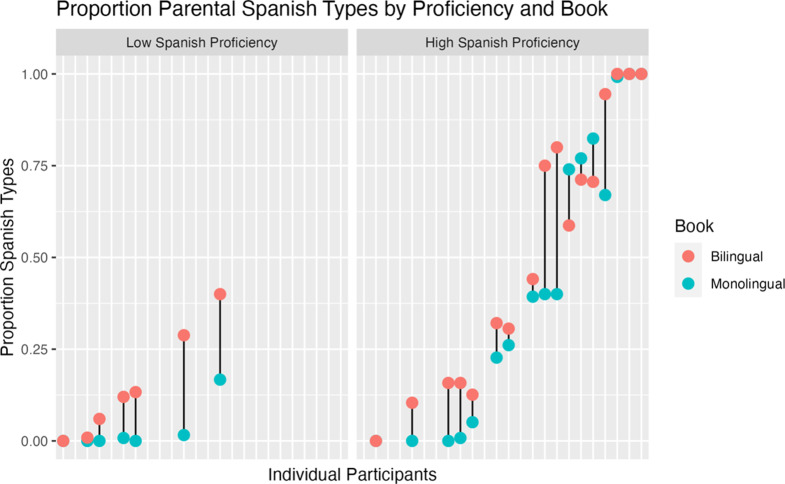
Proportion of Spanish word types by proficiency and book. Proportion of Spanish types used by each participant when sharing the English-Spanish bilingual book (red dots) versus the English monolingual book (blue dots), grouped by participants who had lower Spanish proficiency (*n* = 7) (left panel) and higher Spanish proficieny (*n* = 17) (right panel). The black lines connecting the red and blue dots illustrate the difference in the proportion of Spanish tokens between books for each participant. Figure 5. Long description.

Similarly, there were no significant differences between the higher (*Mdn* = .048) and lower Spanish proficieny (*Mdn* = .133) groups in the difference score for proportion of Spanish word types, *W* = 38.5, *p* = 0.19, CI: 95% [−0.25, 0.04], Cliff’s delta = −.3. Figure 5 shows the same pattern as Figure 4, where a majority of participants, regardless of proficiency group, used a higher proportion of Spanish types when sharing the English-Spanish bilingual book than the English monolingual book.

**Figure 6.**
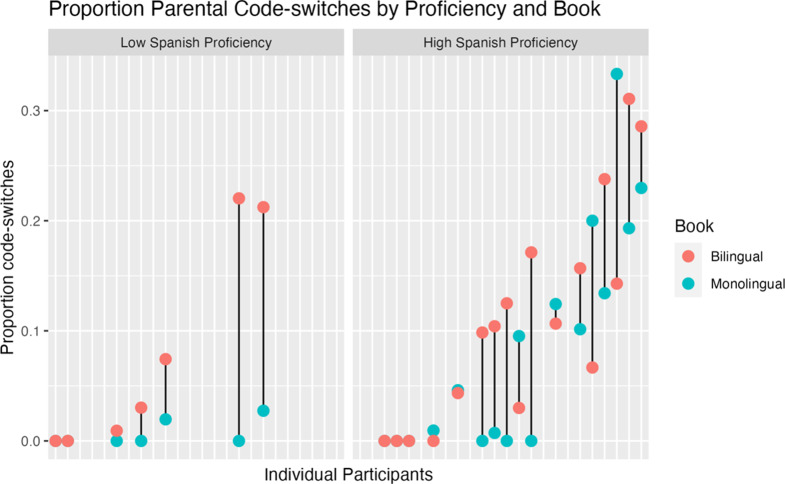
Proportion code-switched utterances by proficiency and book. Proportion of code-switched utterances used by each participant when sharing the English-Spanish bilingual book (red dot) versus the English monolingual book (blue dot), grouped by participants who had lower Spanish proficieny (left panel) (*n* = 7) and higher Spanish proficieny (*n* = 17) (right panel). The black lines connecting the red and blue dots illustrate the difference in the proportion of Spanish tokens between books for each participant. Figure 6. Long description.

As with the other measures, there were no significant differences between the higher (*Mdn* = 0.00) and lower Spanish proficieny (*Mdn* = .03) groups in the difference score for proportion of code-switches *W* = 45, *p* = 0.37, CI: 95% [−0.11, 0.04], Cliff’s delta = −.2 (see Figure 6). Finally, we re-ran these analyses looking at the potential effect of parental English proficiency, and again found no effect of proficiency on any of the differences reported in our main analyses.

## 5. Discussion

This study compared Latine parents’ extra-textual talk when sharing an English-Spanish bilingual book and an English monolingual book. As predicted, parents used a higher proportion of Spanish when sharing the bilingual book than when sharing the English monolingual book, including more Spanish words in total and more different Spanish words. Interestingly, this did not come at the expense of using English, as there were no differences in the amount or diversity of English words across book types. Parents also used more – though not a higher proportion of – code-switched utterances when sharing the bilingual book than when sharing the English monolingual book. These differences in parents’ use of Spanish and code-switching did not significantly differ as a function of their language proficiency. These findings suggest that bilingual books with text in Spanish and English support Latine parents’ use of Spanish in extra-textual talk relative to books with only English text.

Our findings are consistent with previous studies suggesting that children’s books support parents’ extra-textual talk. Studies with monolingual families have shown that parents use talk that is more lexically diverse and syntactically complex during book-sharing than in other contexts (Hoff-Ginsberg, 1991), and one study showed that the syntactic complexity of parents’ extra-textual talk correlates with the syntactic complexity of the text of the book (Noble et al., 2018). In the current study, parents used more different words in Spanish in their extra-textual talk when reading a book with text in English and Spanish than a book without text in Spanish, as measured by the number of different word types. One interpretation of these findings is that the Spanish text in the bilingual book provided parents examples of more different words in Spanish than those they would use without the text’s support, thus increasing lexical diversity in their extra-textual talk. This is consistent with the idea that the text in children’s books can influence the characteristics of parents’ extra-textual talk and suggests this is specific to the language(s) in the text, a finding with important implications for understanding the effects of book-sharing in both monolingual and bilingual families.

However, we did not find a significant difference between book types in the ratio of Spanish word types to word tokens (type-token ratio) – a more conservative measure of lexical diversity (Hoff-Ginsberg, 1991; Malvern et al., 2004). Because the number of different words people use tends to increase as a function of the total number of words they say, it is possible that the difference in the number of different words in Spanish between books was simply a result of parents using more Spanish overall when sharing the English-Spanish bilingual book, rather than being an independent effect of the book text on lexical diversity. However, there are also well-documented problems with using type-token ratio when comparing samples of different lengths, given that type-token ratio decreases as the total number of tokens in a sample increases (Montag et al., 2018). Thus, to further address this question, future studies could investigate the effects of book type on lexical diversity by comparing samples of equal sizes, particularly if samples from multiple book-sharing sessions are collected.

Parents also engaged in more code-switching when sharing the English-Spanish bilingual book than the English monolingual book. First, and not surprisingly, parents were more likely to read in both languages when sharing the English-Spanish bilingual book than when sharing the English monolingual book. In addition, parents used more code-switches in their extra-textual talk when sharing the bilingual than the English monolingual book. This may be because the bilingual text made both languages more accessible, prompting parents to use both languages in their extra-textual talk. It is also possible that seeing bilingual text in a published book was viewed as a signal that mixing languages was acceptable in this context, licensing parents to engage in more code-switching. Alternatively, given that we did not find a significant increase in the *proportion* of code-switches relative to the number of utterances, it is possible that differences in code-switches simply resulted from an overall increase in parental talk between book types. Given previous research showing that bilingual book-sharing strategies, such as embedding words from the non-dominant language within passages in the dominant language or labelling words in both languages, can be effective for supporting bilingual vocabulary development (Bosma et al., 2023; Read et al., 2020), parents’ increased use of code-switching when sharing bilingual books could lead to enhanced comprehension and vocabulary learning for bilingual children. Future studies could directly investigate this possibility.

The current findings are partially consistent with those of recent studies with French-English bilinguals in Montreal. In particular, Gonzalez-Barrero et al. (2021) found that bilingual parents prefer to read in the language in which they are most proficient. And Quirk et al. (2024) found that bilingual parents used their non-dominant language more when sharing both bilingual books and books in their non-dominant language than when sharing books in their dominant language. Here, we found that Latine parents with higher Spanish proficiency used more Spanish overall than parents with lower Spanish proficiency. However, we also found that parents used more Spanish when sharing an English-Spanish bilingual book than an English-only book regardless of proficiency, suggesting the Spanish text influenced Latine parents’ language use above and beyond their language proficiency or dominance. One likely explanation for the different findings between this and previous studies is the sociolinguistic context in which these studies took place. Unlike in Montreal, where English and French hold similar societal value, English is the higher-status language in the U.S., with Spanish still stigmatised in many U.S. contexts (Lippi-Green, 2012). We therefore expected that parents would use more Spanish when sharing English-Spanish bilingual books than when sharing English-only books, regardless of language proficiency, because the bilingual books would act as a cue that using Spanish – the minoritised language – was acceptable and even encouraged. An open question is whether Latine parents would use more or less Spanish when sharing an English-Spanish bilingual book relative to a Spanish-only book. Based on the current findings, we would expect no differences in parents’ Spanish language use between these two contexts. On the other hand, we would predict differences in parents’ use of English, with parents using more English when sharing the English-Spanish bilingual book than the Spanish-only book. Additionally, it is possible that parental language proficiency or dominance would moderate these findings. Independently of what these additional comparisons might find, the current findings suggest bilingual books are at least one way of supporting Latine parents’ home language use.

One possibility is that the increased use of Spanish when sharing the English-Spanish bilingual book was in part due to parents’ cultural and sociolinguistic connection to the language (Chappell & Faltis, 2007; Darr et al., 2020). Studies have shown that encountering Spanish in other forms of media, like news articles, television, and radio, can prime Latine individuals’ cultural identity (Darr et al., 2020; Kerevel, 2011). Thus, it is possible that seeing the Spanish text in children’s books would prime Latine parents’ sociolinguistic identity and increase their motivation to use Spanish when reading with their child. For example, when asked why they preferred the English-Spanish bilingual book, one parent commented: “It is important to me that we teach him Spanish because it’s like my grandpa spoke just Spanish growing up, but wanted his kids to just learn English because they were first-generation, and now they don’t know any Spanish.” Echoing the same sentiments as other participants, this parent emphasises the familial and cultural ties that come with sharing books with Spanish text. Future research could investigate whether sharing bilingual books or books in the minoritised language evokes parents’ cultural identities and the effect this has on their language use during book-sharing interactions.

## 6. Limitations

One limitation of this study is the small number of participants, and in particular of participants with lower proficiency in Spanish or English. Given that we did not originally intend to examine effects of language proficiency or dominance and that the study was not powered with this in mind, our finding that language proficiency did not moderate the effect of book type in this low-powered contrast should be considered exploratory and investigated in future research. Moreover, although we measured parents’ proficiency in English and Spanish, we did not measure language dominance, which may be a more powerful moderating variable in this case. In addition, we compared English-Spanish bilingual books to English-only books, but not to Spanish-only books; it is possible that comparing parents’ language use across English-Spanish bilingual, English-only and Spanish-only books would have revealed a moderating effect of language dominance or proficiency, as has been found in previous studies (Quirk et al., 2024). Finally, the participants in our study are not representative of all Latine families in the U.S., much less of all bilingual families, as many bilingual families, including some bilingual Latine families, speak languages other than Spanish. Future studies with larger and more diverse samples of bilingual families could explore how other socio-demographic characteristics, such as country of origin, language dominance, socioeconomic status, and sociolinguistic context, might influence parents’ language use when sharing bilingual and monolingual books.

## 7. Implications

Findings from the current study have important implications for understanding how books can support the development of bilingual children’s home language, specifically in contexts in which such languages are minoritised such as among Latine families in the U.S. Data from the current study, as well as from larger surveys (Avelar et al., 2024), suggests that Latine parents are more likely to read with their children in English, and that this is the case even among parents who predominantly use Spanish. This pull towards reading in English is likely to have multiple causes, but one of them may be the greater availability of English-language books. Book-sharing advice to families who speak minoritised languages often focuses on ways in which parents can share books in the home language even when the text is in English, such as by talking about the pictures or the story in their home language. Yet our findings suggest the text of the book provides a scaffold that facilitates bilingual parents’ use of their home language in ways that expose children to more words and more unique words – just like monolingual books do for monolingual parents. In addition, parents may use bilingual books as an opportunity to engage in bilingual practices like code-switching as a language-teaching strategy. Indeed, parents in our study commented on the benefits of the bilingual books. One parent commented (about the English-Spanish bilingual book): “If I start reading books like that to her, then I feel like her Spanish is going to be more developed and it’s not going to be forgotten.” This parent then added: “And I feel like reading it to her in Spanish right after reading something to her in English… she’ll know what I’m saying, she’ll like connect the dots almost.” Thus, although it is of course possible for parents to share books without reading the text, and this type of book-sharing still brings many benefits, bilingual books may provide unique opportunities for parents to use the home language and to support their children’s bilingual language development. Altogether, these findings highlight the importance of increasing families’ access to children’s books in their home or heritage language, which could take the form of bilingual books.

## 8. Conclusion

The results from this study add to the evidence suggesting that sharing books with young children can help support bilingual language development in early childhood. We show that English-Spanish bilingual books increase Latine parents’ use of Spanish in extra-textual talk, increase the lexical diversity of their Spanish extra-textual talk, and increase their use of code-switching, all of which could help support Latine children’s development of the home language. Given that developing skills in both languages is important for bilingual children’s academic success and that many immigrant parents desire that their children learn and maintain the heritage language, making bilingual books more readily available to these families may have important benefits.

## Footnotes

1 We use the gender-inclusive term Latine to refer to individuals who have Latin American heritage. The term Hispanic is used only if it was used as such in its original source.

## Long descriptions

### Table 1. Long description

The table presents data for n equals 24 participants across three main categories.

1. Child Gender

- Male: 10 participants (41.7 percent)

- Female: 14 participants (58.3 percent)

2. Parental Education

- Less than H S or some H S: 4 participants (16.7 percent)

- H S equivalent or Some college: 4 participants (16.7 percent)

- College degree: 11 participants (45.9 percent)

- Graduate degree: 4 participants (16.7 percent)

- Missing: 1 participant (4.2 percent)

3. Parent language proficiency

- Lower Spanish proficiency: 7 participants (29 percent)

- Higher Spanish proficiency: 17 participants (71 percent)

- Lower English proficiency: 3 participants (12.5 percent)

- Higher English proficiency: 21 participants (87.5 percent)

Note: Lower proficiency is defined as speaking the language not well at all, slightly well, or moderately well. Higher proficiency is defined as speaking the language very or extremely well.

Navigate back to Table 1..

### Table 2. Long description

The table is organized into five columns. The first column lists the linguistic metrics, while the remaining four columns are grouped under two main headers.

Header Group 1: English monolingual books.

- Column 2: My Friends.

- Column 3: How Do I Love You?

Header Group 2: English-Spanish bilingual books.

- Column 4: My Friends Mis Amigos.

- Column 5: How Do I Love You? ¿Como te quiero?

Data rows for each metric:

- Number of sentences: 17 for both monolingual books; 34 for both bilingual books.

- Mean sentence length in words: 9.06 for My Friends; 8.83 for How Do I Love You?; 8.68 for My Friends Mis Amigos; 8.31 for How Do I Love You? ¿Como te quiero?.

- Total number of words: 154 for My Friends; 159 for How Do I Love You?; 295 for My Friends Mis Amigos; 299 for How Do I Love You? ¿Como te quiero?.

- Number of unique root words: 47 for My Friends; 67 for How Do I Love You?; 99 for My Friends Mis Amigos; 134 for How Do I Love You? ¿Como te quiero?.

Navigate back to Table 2..

### Table 3. Long description

The table consists of two columns: Primary language in which parent and N percentage. It is divided into two main sections.

Section 1: Speaks to child.

- Mostly or only English: 7 (29.1 percent).

- Mostly or only Spanish: 10 (41.6 percent).

- Equally English and Spanish: 7 (29.1 percent).

Section 2: Reads to child.

- Mostly or only English: 11 (45.8 percent).

- Mostly or only Spanish: 4 (16.6 percent).

- Equally English and Spanish: 9 (37.5 percent).

Navigate back to Table 3..

### Table 4. Long description

The table is divided into two main columns: English monolingual book and English-Spanish bilingual book. Each main column is subdivided into Median (M d n), Inter-quartile Range, and Range.

Under Word tokens:

- English: Monolingual M d n 74.5, Range 0 to 623; Bilingual M d n 102.5, Range 0 to 1279.

- Spanish: Monolingual M d n 7, Range 0 to 350; Bilingual M d n 34.5, Range 0 to 488.

- Proportion (Spanish tokens over total): Monolingual M d n 0.20; Bilingual M d n 0.30.

Under Word types:

- English: Monolingual M d n 49, Range 0 to 187; Bilingual M d n 59, Range 0 to 234.

- Spanish: Monolingual M d n 3, Range 0 to 118; Bilingual M d n 26.5, Range 0 to 154.

- Proportion (Spanish types over total): Monolingual M d n 0.20; Bilingual M d n 0.31.

Under Code-switches:

- Total: Monolingual M d n 1, Range 0 to 34; Bilingual M d n 7.5, Range 0 to 34.

- Proportion (switched utterances over total): Monolingual M d n 0; Bilingual M d n 0.09.

Navigate back to Table 4..

### Figure 1. Long description

The Y axis is labeled Proportion of Spanish Tokens and ranges from 0.00 to 1.00 in increments of 0.25. The X axis is labeled Book and contains two categories: Monolingual on the left and Bilingual on the right.

* The Monolingual box is colored salmon pink. Its base is at 0.00, the median line is at approximately 0.20, and the top of the box is at approximately 0.78. The upper whisker extends to 1.00.

* The Bilingual box is colored teal. Its base is at approximately 0.05, the median line is at approximately 0.30, and the top of the box is at approximately 0.84. The upper whisker extends to 1.00.

Individual data points for each participant are represented by black dots. Light gray lines connect the dots between the Monolingual and Bilingual conditions. Most lines show a slight upward slope, indicating an increase in the proportion of Spanish tokens in the Bilingual book compared to the Monolingual book for those individuals, though several participants remain at or near 0.00 or 1.00 in both conditions.

Navigate back to Figure 1..

### Figure 2. Long description

The vertical y-axis is labeled Proportion of Spanish Types and ranges from 0.00 to 1.00 in increments of 0.25. The horizontal x-axis is labeled Book and contains two categories.

On the left, the Monolingual condition features a red box plot. The median line is positioned at approximately 0.20. The box extends from 0.00 to roughly 0.70. Whiskers extend to the maximum value of 1.00 and the minimum of 0.00.

On the right, the Bilingual condition features a teal box plot. The median line is higher, at approximately 0.32. The box extends from about 0.12 to 0.72. Whiskers extend to 1.00 and 0.00.

Individual data points for participants are plotted as black dots over both boxes. Light gray lines connect the dots between the Monolingual and Bilingual conditions. Most lines show an upward slope, indicating an increase in the proportion of Spanish word types for individual participants when moving from the Monolingual to the Bilingual book, though several participants remain at 0.00 or 1.00 in both conditions.

Navigate back to Figure 2..

### Figure 3. Long description

The vertical Y axis is labeled Proportion Code-switched Utterances and ranges from 0.0 to 0.3. The horizontal X axis is labeled Book and contains two categories.

1. Monolingual (left): A red box plot shows a median near 0.01, with an inter-quartile range extending up to approximately 0.11. Whiskers reach from 0.0 to roughly 0.23. Individual black dots are clustered heavily near 0.0, with several outliers extending up to 0.33.

2. Bilingual (right): A teal box plot shows a higher median near 0.09, with an inter-quartile range from approximately 0.01 to 0.16. Whiskers reach from 0.0 to roughly 0.31. Individual black dots are more evenly distributed across the range.

Light gray lines connect individual dots from the Monolingual column to the Bilingual column. Most lines show an upward slope, indicating an increase in code-switching for most participants when moving from the monolingual to the bilingual book condition, though a few lines show a decrease or remain flat near zero.

Navigate back to Figure 3..

### Figure 4. Long description

The dot plot is divided into two panels. The vertical y-axis is labeled Proportion Spanish Tokens and ranges from 0.00 to 1.00. The horizontal x-axis is labeled Individual Participants. A legend on the right indicates red dots for Bilingual books and blue dots for Monolingual books.

* Left Panel: Low Spanish Proficiency. This panel shows seven participants. Most participants have very low proportions near 0.00 for both books. Two participants show a notable increase when using the bilingual book, with vertical black lines connecting a blue dot near 0.00 to a red dot at approximately 0.23, and another connecting a blue dot at 0.24 to a red dot at 0.40.

* Right Panel: High Spanish Proficiency. This panel shows seventeen participants. Data points are generally higher on the y-axis compared to the left panel. For many participants, the red bilingual dot is higher than the blue monolingual dot, indicated by vertical black lines. In the upper right of this panel, several participants reach a proportion of 1.00 for both book types, where the red and blue dots overlap or sit adjacent at the top of the scale.

Navigate back to Figure 4..

### Figure 5. Long description

The graph consists of two side-by-side panels with a shared y-axis labeled Proportion Spanish Types ranging from 0.00 to 1.00. The x-axis is labeled Individual Participants. A legend on the right indicates that red dots represent the Bilingual book and blue dots represent the Monolingual book.

* Left Panel (Low Spanish Proficiency): Shows data for 7 participants. Most participants have very low proportions of Spanish types (near 0.00) for both books. Two participants show a notable increase when using the bilingual book, with red dots reaching approximately 0.30 and 0.40, while their corresponding blue dots remain below 0.20. Black vertical lines connect the dots for each individual, showing a consistent upward trend from monolingual to bilingual books.

* Right Panel (High Spanish Proficiency): Shows data for 17 participants. The data points are generally higher on the y-axis compared to the left panel. On the far right of this panel, several participants reach a proportion of 1.00 for both book types. For most participants in this group, the red dots (Bilingual) are higher than the blue dots (Monolingual), indicated by the vertical black lines. However, the gap between the two book types varies significantly across individuals, with some showing nearly identical proportions for both.

Navigate back to Figure 5..

### Figure 6. Long description

A two-panel dot plot with the y-axis labeled Proportion code-switches ranging from 0.0 to 0.3 and the x-axis labeled Individual Participants. A legend on the right identifies red dots as Bilingual and blue dots as Monolingual.

* Left Panel: Low Spanish Proficiency. Contains 7 participants. Most participants show very low proportions near 0.0. Two participants on the right of this panel show a significant increase in code-switching for the bilingual book, with red dots near 0.2 and blue dots near 0.0, connected by long vertical black lines.

* Right Panel: High Spanish Proficiency. Contains 17 participants. Data shows a general upward trend from left to right. While some participants remain near 0.0, many show higher proportions of code-switching. In several cases, the blue monolingual dot is higher than the red bilingual dot, particularly for the participant with the highest overall proportion, where the blue dot is above 0.3 and the red dot is just above 0.1. Vertical black lines connect the two book types for each individual, illustrating the variance in switching behavior based on the text provided.

Navigate back to Figure 6..
